# CRISPR/dual-FRET molecular beacon for sensitive live-cell imaging of non-repetitive genomic loci

**DOI:** 10.1093/nar/gkz752

**Published:** 2019-08-31

**Authors:** Shiqi Mao, Yachen Ying, Xiaotian Wu, Christopher J Krueger, Antony K Chen

**Affiliations:** 1 Department of Biomedical Engineering, College of Engineering, Peking University, Beijing 100871, China; 2 School of Life Sciences, Peking University, Beijing 100871, China; 3 Wallace H Coulter Department of Biomedical Engineering, Georgia Institute of Technology, Atlanta, GA 30332, USA

## Abstract

Clustered regularly interspaced short palindromic repeats (CRISPR)-based genomic imaging systems predominantly rely on fluorescent protein reporters, which lack the optical properties essential for sensitive dynamic imaging. Here, we modified the CRISPR single-guide RNA (sgRNA) to carry two distinct molecular beacons (MBs) that can undergo fluorescence resonance energy transfer (FRET) and demonstrated that the resulting system, CRISPR/dual-FRET MB, enables dynamic imaging of non-repetitive genomic loci with only three unique sgRNAs.

## INTRODUCTION

The nuclease-deactivated mutant of the clustered regularly interspaced short palindromic repeats (CRISPR)-associated protein 9 (dCas9) retains the ability to bind a specific target genomic locus through association with Cas9 cognate single-guide RNA (sgRNA), thus spurring the development of CRISPR-based approaches for noninvasive genomic imaging (1–10). However, most approaches have employed dCas9 or sgRNA modified to carry fluorescent proteins (FPs), which lack sufficient brightness and photostability necessary for continuous imaging. Organic dyes (fluorophores), compared to FPs, are generally brighter and more photostable. Additionally, the fluorescence of many fluorophores can be significantly reduced when situated in close spatial proximity to a compatible quencher. Accordingly, various fluorogenic probes have been developed based on fluorophore-quencher pairs to enable detection of specific biomolecules in living cells without the need to wash away unbound probes. One commonly used fluorogenic probe is the molecular beacon (MB) (11), a class of stem-loop-forming oligonucleotide probes containing a fluorophore and a quencher at the two termini that has been used extensively for live-cell RNA imaging (12–14). Prior to activation, MBs exhibit a low fluorescence signal as the complementary short-arm sequences at the two termini self-anneal to form a stable stem duplex that holds the fluorophore and the quencher in close proximity. Hybridization of target RNA to the loop domain disrupts the stem duplex, resulting in separation of the quencher from the fluorophore to restore MB fluorescence.

**Figure 1.**
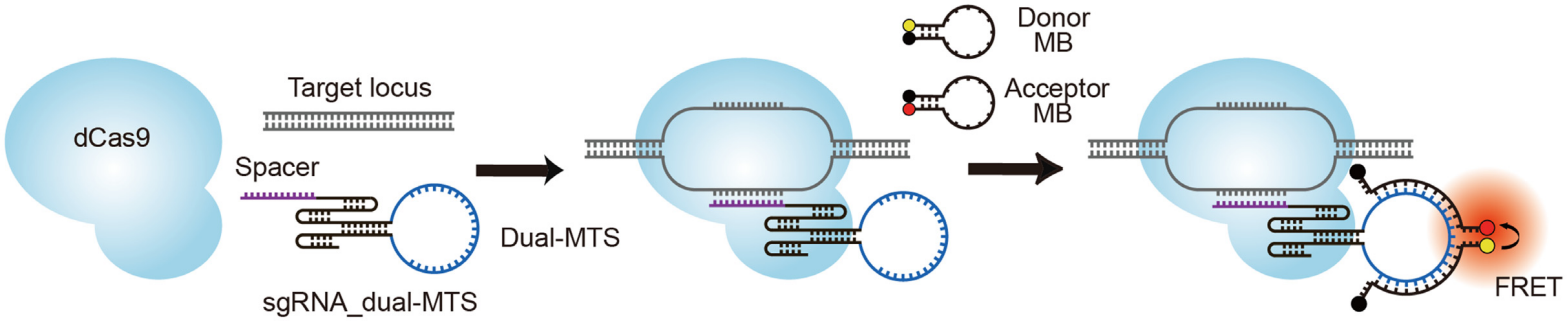
Schematic of CRISPR/dual-FRET MB for labeling a specific locus. To illuminate a specific genomic locus, dCas9 and sgRNA_dual-MTS were first co-expressed to target the locus in cells. This is followed by delivery of both donor and acceptor MBs (dual-FRET MBs) that, upon hybridization to the same dual-MTS, can result in FRET to illuminate the target locus.

Recognizing the utility of sgRNAs for genomic labeling and the useful fluorogenic properties of MBs upon RNA hybridization, our laboratory has recently combined CRISPR and MB systems to create a genomic imaging platform termed CRISPR/MB (9), which consists of dCas9, an MB, and an sgRNA engineered to harbor a unique MB target sequence not found in the human genome (MTS). To illuminate a specific genomic locus, dCas9 and the modified sgRNA are first co-expressed to target the locus in cells, followed by delivery of MBs that, upon hybridization to the MTS, can illuminate the target locus. Through imaging of repetitive elements within telomeres, we demonstrated that CRISPR/MB is more efficient and sensitive than conventional approaches utilizing telomere repeat binding proteins fused to an FP. In this study, we sought to further refine the CRISPR/MB system by modifying the sgRNA to harbor two distinct MB target sequences (sgRNA_dual-MTS) for two distinct MBs whose fluorophores form a fluorescence resonance energy transfer (FRET) pair (Figure 1), a strategy termed dual-FRET MB (15,16). Since FRET only occurs when the two MBs hybridize to the same RNA, dual-FRET MBs are expected to avoid background signals resulting from imperfect quenching and nonspecific protein binding of single MBs. Specific experiments were performed to assess the capacity of our proposed system, named CRISPR/dual-FRET MB, for live-cell imaging of non-repetitive regions within genomic loci using widefield fluorescence microscopy.

## MATERIALS AND METHODS

### Cloning dCas9 expression constructs

To generate BFP/dCas9-C1, which encodes BFP (a transfection control) and the nuclease-deactivated *Streptococcus pyogenes* Cas9 (dCas9) under the control of separate promoters, telomere-targeting sgRNA (sgTelo) was removed from sgTelo/BFP/dCas9-C1 described previously (9) using AseI, followed by self-ligation of the resulting vector. To generate pCMV-dCas9-EGFP-C1, which encodes dCas9 fused to EGFP (dCas9-EGFP), the coding region of dCas9-EGFP was first PCR amplified from pSLQ1658-dCas9-EGFP (Addgene plasmid #51023) (4) using forward primer 5′-GCTACCGGTCGCCACCATGGTGCCCAAAAAGAAGAGGAAAGTGGACAAGA-3′ and reverse primer 5′-GTAGAATTCTTACTTGTACAGCTCGTCCATGCCGAG-3′. The PCR product was then inserted into the pmTagBFP2-C1 vector (a gift from Prabuddha Sengupta, Janelia Research Campus, USA) digested with AgeI and EcoRI to excise mTagBFP2.

### Cloning sgRNA expression constructs

#### For labeling non-repetitive regions

A plasmid harboring a U6-sgRNA_dual-MTS cassette was custom-made by Beijing Genomics Institute (Beijing, China) and was used to generate a backbone plasmid for each sgRNA targeting a non-repetitive region. Specifically, the spacer sequence of sgRNA_dual-MTS was replaced with a spacer sequence designed to hybridize to a unique region within the *MUC4, MUC1*, or IGR locus, or a nonsense spacer sequence (control), by PCR-mediated site-directed mutagenesis (See Supplementary Table S1 for sgRNA scaffold information, Supplementary Table S2 for spacer/target region information, and Supplementary Table S3 for primer information). Thereafter, the PCR product of the U6-sgRNA_dual-MTS cassette from each backbone plasmid (Forward primer: 5′-ACTGCTGTCGACAATGCGTCGAGATCCAATTAGTTAT-3′; reverse primer: 5′-ACCTGCGGATCCCTCGAGTTGTGAGCGGATAACAATTTCACAC-3′) was inserted into the pGEM-11zf(+) vector digested with SalI and BamHI to create an sgRNA expression construct for each sgRNA. From the resulting constructs, a set of multiplexed sgRNA expression constructs containing different numbers of unique U6-sgRNA_dual-MTS cassettes (See Supplementary Table S4) were generated using the protocol described previously by van den Bogaard and Tyagi (17). For *MUC4*, a multiplexed sgRNA expression construct encoding three unique sgRNAs lacking the dual-MTS (sgMUC4) with the same spacers as the 3 sgMUC4_dual-MTS of Set I (See Supplementary Table S1, Table S2 and Table S4) were also generated using the same protocol, with the parental constructs obtained by PCR-mediated site-directed mutagenesis to remove the dual-MTS and additional sequences flanking the dual-MTS using forward primer 5′-GAAAAAGTGGCACCGAGTCGGTGCTTT-3′ and reverse primer 5′-AAGTTGATAACGGACTAGCCTT-3′.

#### For labeling repetitive regions

To generate pSLQ1661-sgMUC4-E3(F+E)/BFP, a construct that encodes an unmodified sgRNA (lacking dual-MTS) with a spacer sequence complementary to a repetitive sequence proximal to the non-repetitive regions of the *MUC4* gene (*MUC4*-NRR) as described above (sgRNA-rep (Proximal to *MUC4*-NRR), see Supplementary Table S1 and Table S5), mTagBFP2 was PCR amplified from the pmTagBFP2-C1 vector using forward primer 5′-AGCGCTACCGGTCGCCACCATG-3′ and reverse primer 5′-ACTGCAGAATTCTTAATTAAGCTTGTGCCCCAGTTTGC-3′ and then inserted into pSLQ1661-sgMUC4-E3(F+E) (Addgene plasmid # 51025) (4) digested with AgeI and EcoRI to excise out mCherry. To generate constructs that encode sgRNAs targeting other repetitive regions (See Supplementary Table S5), the U6-sgMUC4-E3(F+E) cassette of pSLQ1661-sgMUC4-E3(F+E)/BFP was first PCR amplified using forward primer 5′-ACTGCTGTCGACTTTGGTTAGTACCGGGCCCGCTCTA-3′ and reverse primer 5′-CTAATGGATCCTAGTACTCGAGAAA-3′, followed by subcloning the PCR product into the pGEM-11zf(+) vector digested with SalI and BamHI. The spacer sequence in the subcloning vector was replaced with the spacer of each sgRNA by PCR-mediated site-directed mutagenesis (See Supplementary Table S6 for primer information). The region that contains the U6-sgRNA cassette in the resulting plasmid was then cloned back into pSLQ1661-sgMUC4-E3(F+E)/BFP digested with XbaI and BamHI to excise out the U6-sgMUC4-E3(F+E) cassette.

### Synthesis of MBs

The donor MB was labeled with an Iowa Black FQ quencher at the 5′-end and an ATTO550 fluorophore at the 3′-end and has the sequence: 5′-mCmUmCmAmG*mC*mG*mU*mA*mA*mG*mU*mG*mA*mU*mG*mU*mC*mG*mU*mG*mA*mCmUmGmAmG-3′. The acceptor MB was labeled with an ATTO647N fluorophore at the 5′-end and an Iowa Black RQ quencher at the 3′-end, and has the sequence: 5′-mCmUmUmCmG*mU*mC*mC*mA*mC*mA*mA*mA*mC*mA*mC*mA*mA*mC*mU*mC*mC*mU*mGmAmAmG-3′ (Underlined letters indicate the MB stem, m represents 2′-*O*-methyl RNA modification; * represents phosphorothioate linkage modification). The MB sequences are designed to avoid hybridization with endogenous RNAs in mammalian cells. All MBs were synthesized by Integrated DNA Technologies (Coralville, IA, USA).

### Cell culture and transfection

HeLa and U2OS cells (American Type Culture Collection) were cultured in Dulbecco's Modified Eagle's Medium (DMEM, Mediatech), supplemented with 10% (vol/vol) FBS (PAN™ Biotech) and 1× GlutaMAX™ (Thermo Fisher) at 37°C, 5% (vol/vol) CO_2_ and 90% relative humidity. Plasmid transfection was performed with FuGENE^®^ 6 (Promega) according to the manufacturer's protocols when cells reached 50–70% confluency in 6-well plate. To image non-repetitive regions using CRISPR/dual-FRET MB, HeLa or U2OS cells were transfected with 600 ng of BFP/dCas9-C1 and 1400 ng of either a single or multiplexed sgRNA expression construct (See Supplementary Table S4). For co-labeling experiments, the cells were transfected with 600 ng of pCMV-dCas9-EGFP-C1, 600 ng of a multiplexed sgRNA expression construct and 600 ng of pSLQ1661-sgRNA-rep. For fluorescence *in situ* hybridization experiments, HeLa cells seeded on 8-well Lab-Tek™ chambered coverglass previously coated with fibronectin were transfected with 60 ng of BFP/dCas9-C1, 60 ng of pSLQ1661-sgRNA-rep (Distal to *MUC4*-NRR) and 60 ng of pSLQ1661-sgRNA-rep (Proximal to *MUC4*-NRR). All experiments were performed with cells at passage numbers between 5 and 25.

### Delivery of MBs

MBs were delivered into cells by nucleofection/microporation according to methods described previously (18–20), with modifications. In brief, cells grown to 70% confluency were trypsinized, washed with 1× PBS and pelleted, followed by resuspension in 11 μl of 1× PBS containing equal amounts of donor and acceptor MBs to obtain final cell concentration of 5,000 cells per μl and a final MB concentration of 2 μM for each MB. Thereafter, 10 μl of the cell mixture (roughly 50,000 cells) were nucleofected using the Neon^®^ Transfection System with the parameters set at 1005 V with a 35 ms pulse width and two pulses total for HeLa cells and 1230 V with a 10 ms pulse width and four pulses total for U2OS cells. Following nucleofection and three washes in culture medium to remove free MBs, the cells were seeded on 8-well Lab-Tek^TM^ chambered coverglass previously coated with fibronectin.

It should be noted that other cellular delivery methods, such as streptolysin-O (15), have also been used to deliver MBs and therefore are expected to be effective in delivering the donor and acceptor MBs used in this study.

### Fluorescence *in situ* hybridization

HeLa cells containing dCas9, sgRNA-rep (Distal to *MUC4*-NRR) and sgRNA-rep (Proximal to *MUC4*-NRR) (See Supplementary Table S5) were subjected to fluorescence *in situ* hybridization (FISH) experiments as previously described (3,9), with modifications. In brief, cells were fixed with 4% paraformaldehyde (PFA) in 1× PBS for 30 min at room temperature, followed by two washes with 1× PBS. Thereafter, the cells were permeabilized with 0.5% (vol/vol) NP-40 in 1× PBS for 10 min and then washed once with 1× PBS. Following a 5-minute incubation of the cells in 1× PBS, the cells were incubated in hybridization buffer (1% (vol/vol) Tween^®^ 20, 10% (vol/vol) dextran sulfate, 50% (vol/vol) formamide, 500 ng/ml Salmon sperm DNA in 2× saline-sodium citrate (SSC) buffer) containing 100 nM FISH probes (5′-CCGTCAATTTTACTTTATGTCT-ATTO488-3′) and 100 nM FISH probes (5′-TAMRA-CCTCCTGTCACCGAC-3′) targeting the repetitive regions distal to *MUC4*-NRR and proximal to *MUC4*-NRR, respectively, in a humidified chamber at 37°C for 24 h. The cells were washed in wash buffer (2× SSC, 10% (vol/vol) formamide) followed by 2× SSC, 1× SSC and 0.2× SSC to remove unhybridized probes and then incubated in 1× PBS prior to imaging. It should be noted that interactions between dCas9 and sgRNA lead to unwinding of the DNA duplex, allowing the FISH probes to hybridize to the strand not bound to the sgRNA spacer without the need for high-temperature heating.

### Fluorescence microscopy

Fluorescence microscopy experiments were performed on an Olympus IX 83 motorized inverted fluorescence microscope equipped with the CellTIRF-4Line system, a 100× UPlanSApo 1.4NA objective lens, a back-illuminated EMCCD camera (Andor), Sutter excitation and emission filter wheels under the control of the CellSens Dimension software. Images of DAPI, EGFP/ATTO488 and TAMRA were acquired using the Olympus MT20 filter set for DAPI, EGFP and TAMRA and images of FRET and ATTO647N were acquired using Chroma filter sets (ET545/25x, ET700/75m, T565lpxr) and (ET620/60x, ET700/75m, T660lpxr), respectively, with the excitation light provided by a X-Cite Series 120 light source housing a Mercury Lamp (EXFO). Simultaneous, dual-color images of EGFP and FRET were acquired using IX3-U-m4TIR-Sbx, and a DV2-cube (ET525/50m, 585dcxr, ET655lp, Photometrics), with the excitation light of EGFP and FRET provided by a 488nm laser line (150 mW) and a 561nm laser line (150 mW), respectively. Three-dimensional (3D) image stacks were acquired with 0.25 μm increments in the z-direction. All images were analyzed using Fiji (21), the AutoQuant deconvolution software (MediaCybernetics), or custom-written MATLAB (Version R2014b 64-bit, Mathworks) programs.

### Identification of single genomic loci

Identification of single genomic loci in HeLa and U2OS cells was performed as described previously (18,19), with minor modifications. In brief, rolling-ball background subtraction (background = 2) was first applied on all 3D images to enhance particulate objects. This was followed by identification of particles using the 3D Laplacian of Gaussian plug-in available for Fiji. After manually setting the threshold to remove low-intensity spots, a region of interest (ROI) was drawn around each nucleus and applied to the filtered stack. The Find Stack Maxima macro plug-in (Exclude Edge Maxima; Noise Tolerance = 0) was then used to identify all local maxima in each slice of the z-stack. To identify which two-dimensional (2D) local maxima were 3D local maxima and to quantify the total number of 3D local maxima, a custom MATLAB program was written to compare the intensity of each local maximum in each slice with the intensity of each pixel within a 5 × 5 × 5 voxel cube centered around the local maximum. Each 3D maximum was considered a single locus and the total number of loci per nucleus was computed.

### 3D colocalization analysis

After determining the 3D coordinates of genomic loci in the dCas9-EGFP and dual-FRET MB images using the methods described above, a custom MATLAB program was employed to identify the colocalization level in 3D as previously described (18,19), with minor modifications. In brief, an MB 3D local maximum was considered to be an MB colocalization event if an EGFP 3D local maximum was found within a 7 × 7 × 7 voxel cube centered around the MB maximum. The colocalization percentage was calculated by dividing the number of MB colocalization events by the total number of MB local maxima.

### Signal-to-noise ratio analysis

2D images were acquired for both dual-FRET MBs and single MBs with one-second exposure time and subjected to signal-to-noise ratio (SNR) analysis, as described previously (22), according the following formula:

$$\begin{equation*}{\rm{SNR}} = \frac{{{{\rm{i}}_{{\rm{spot\ signal,\ maximum}}}} - {{\rm{i}}_{{\rm{background,\ mean}}}}}}{{{{\rm{i}}_{{\rm{background,\ \sigma }}}}}}\end{equation*}$$

where *i*_spot signal,maximum_ refers to the maximum fluorescence intensity of a single genomic locus, *i*_background,mean_ refers to the mean background signal and *i*_background,σ_ refers to the standard deviation of the background signal.

### Single-particle tracking analysis

2D time-lapse images were analyzed to identify the tracks of single genomic loci using the TrackMate plugin of Fiji, as described previously (18,19). In brief, individual peaks and their coordinates were determined by using Laplacian of Gaussian (LoG) detector. Thereafter, peaks that belong to the same track were determined by simple Linear Assignment Problem tracker (LAP). The resulting tracks were then used for the co-movement and diffusion coefficient analyses as described below:

*Co-movement analysis*. For analysis of co-movement between a dCas9-EGFP-labeled spot and a CRISPR/dual-FRET MB-labeled spot, simultaneous dual-color images acquired at 10 frames per second (fps) were used. Within a set of dual-color images, spots in the EGFP channel and in the FRET channel were paired based on the closest spatial distance. Following the assignment of spot pairs, the *x*–*y* coordinates of each spot were determined with LAP parameters set at linking max distance = 0.5 μm; gap-closing max distance = 0; gap-closing max frame gap = 0, followed by cross-correlation analysis of spot pairs in MATLAB based on the following formula as described previously (23):

$$\begin{equation*}{\rm{\rho \ }} = \frac{{\left\langle {{{\boldsymbol{r}}_{EGFP}} \cdot {{\boldsymbol{r}}_{FRET}}} \right\rangle - \left\langle {{{\boldsymbol{r}}_{EGFP}}} \right\rangle \cdot \left\langle {{{\boldsymbol{r}}_{FRET}}} \right\rangle }}{{{{\left[ {\left( {\left\langle {{\boldsymbol{r}}_{EGFP}^2} \right\rangle - {{\left\langle {{{\boldsymbol{r}}_{EGFP}}} \right\rangle }^2}} \right)\left( {\left\langle {{\boldsymbol{r}}_{FRET}^2} \right\rangle - {{\left\langle {{{\boldsymbol{r}}_{FRET}}} \right\rangle }^2}} \right)} \right]}^{1/2}}}}{\rm{\ }}\end{equation*}$$

where ρ is the cross-correlation coefficient, ***r_EGFP_*** and ***r_FRET_*** are the position vectors of the dCas9-EGFP- and CRISPR/dual-FRET MB-labeled spots, respectively. ρ ranges from -1 for completely anticorrelated motions to +1 for completely correlated motions.

*Diffusion coefficient analysis*. For analysis of diffusion coefficients, images acquired at 50 fps were used, with the LAP parameters set at linking max distance = 0.1 μm; gap-closing max distance = 0.4 μm; gap-closing max frame gap = 4. The resulting peaks and their *x*–*y* coordinates were imported into @msdanalyzer written in MATLAB (24) and tracks containing at least 15 time lags (Δτ) were selected for calculating the Mean Square Displacement (MSD). For simplicity, the 2-D diffusion coefficient D_eff_ and the diffusive exponent α of all trajectories were obtained from log-log fit of the following formula:

$$\begin{equation*}< {\rm{MSD}} >= 4{{\rm{D}}_{{\rm{eff}}}}\Delta {\tau ^\alpha }\end{equation*}$$

using the first 25% of total time lags, with a minimum fitting threshold of *R*^2^ > 0.8. Tracks with α < 1.2 represented diffusion while tracks with α > 1.2 represented directed transport.

### Data analysis

Statistical analyses were performed using either two-tailed Student's *t*-test or one-way ANOVA with post hoc testing of pairwise comparisons using Scheffe's test.

## RESULTS AND DISCUSSION

### Assessing the sensitivity of CRISPR/dual-FRET MB for imaging non-repetitive genomic regions

**Figure 2.**
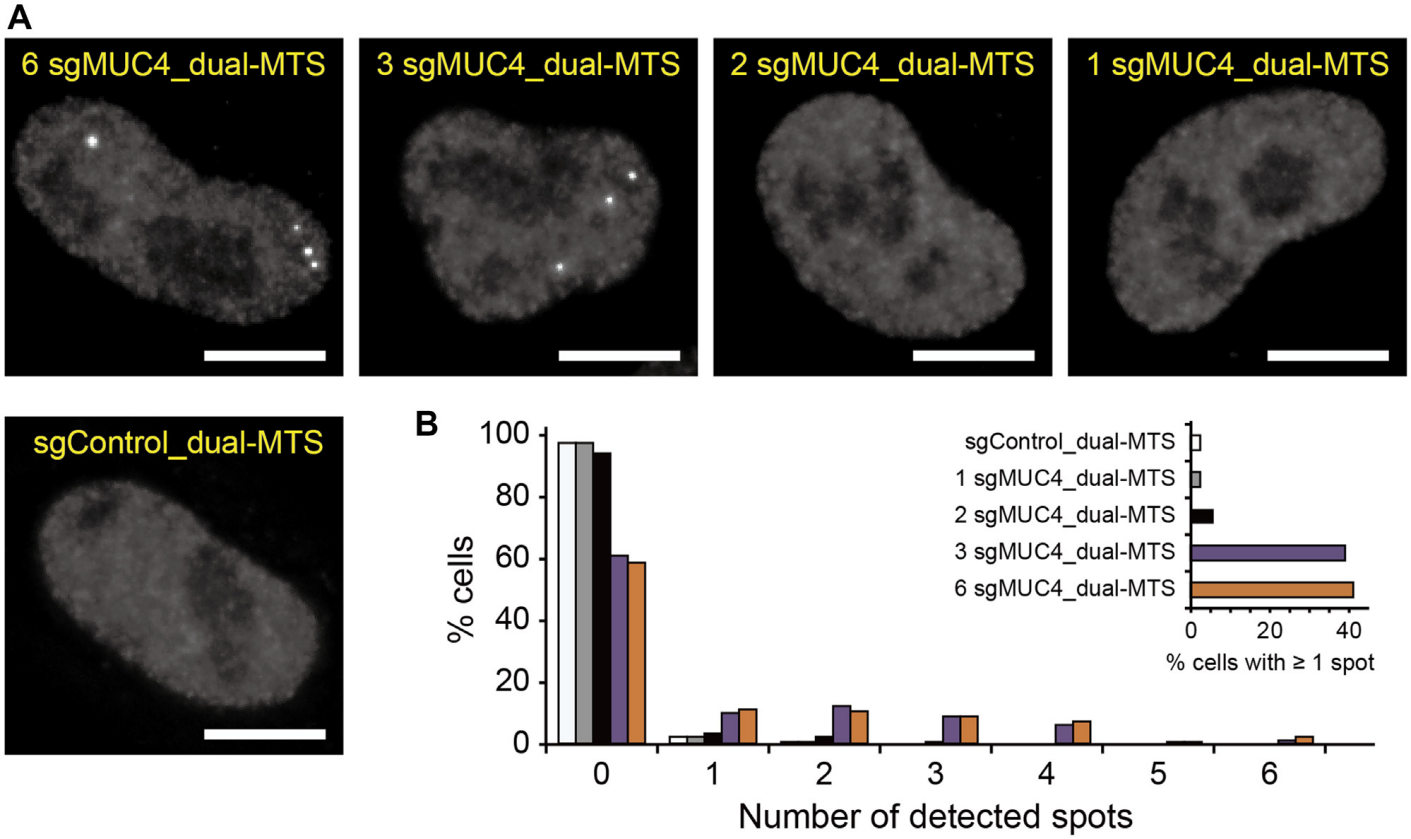
Imaging non-repetitive regions of *MUC4* by CRISPR/dual-FRET MB with different numbers of unique *MUC4*-targeting sgRNAs. HeLa cells expressing dCas9 and either 6, 3, 2 or 1 unique sgMUC4_dual-MTS or sgControl_dual-MTS were nucleofected with dual-FRET MBs. Fluorescence microscopy images were taken at 24 h post-nucleofection. (**A**) Representative maximum intensity projection images of dual-FRET MB signals in fixed cells. Scale bar, 10 μm. (**B**) The distribution of spot number in the cells. The inset shows detection efficiency. *n* = 300 cells from three independent experiments for each condition.

**Figure 3.**
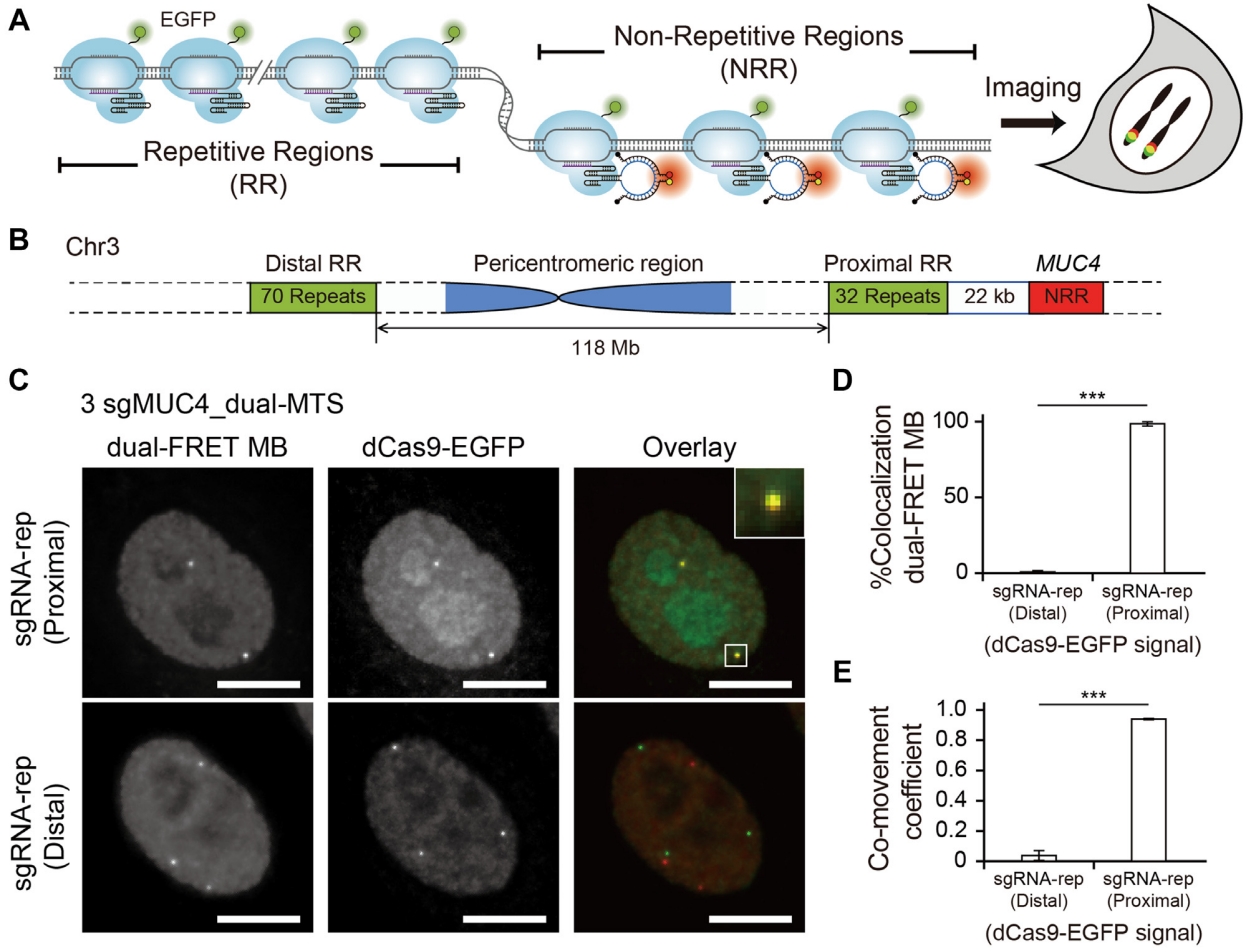
CRISPR/dual-FRET MB can illuminate non-repetitive regions of *MUC4* with 3 unique sgRNAs. HeLa cells expressing dCas9-EGFP, three unique sgMUC4_dual-MTS (Set I, see Supplementary Table S4), and an unmodified sgRNA targeting highly-repetitive genomic regions (sgRNA-rep) on the same chromosome (Chr3) were nucleofected with dual-FRET MBs. (**A**) Schematic of the co-labeling experiment. (**B**) Schematic showing the position of the non-repetitive regions (NRR) and the repetitive regions (RR) proximal or distal to NRR on Chr3 selected for labeling. (**C**) Representative maximum intensity projection images of dual-FRET MB and dCas9-EGFP signals in fixed cells. Inset shows colocalization of the two signals when dCas9-EGFP labels proximal RR through sgRNA-rep. Scale bar, 10 μm. (**D**) The percentage of dual-FRET MB signals colocalizing with dCas9-EGFP signals (%Colocalization dual-FRET MB) representing proximal or distal RR on a cell-by-cell basis (n = 35 cells for proximal RR and 30 cells for distal RR). (**E**) The correlation coefficient between dual-FRET MB and dCas9-EGFP signals (co-movement coefficient) in living cells. 15 trajectories of each signal were analyzed for proximal RR and 18 trajectories of each signal were analyzed for distal RR, both from 12 cells. Note that co-movement coefficient of 1 indicates perfect co-movement. All data represent mean ± S.E. of three independent experiments. Asterisks indicate *P*-values (*** *P* < 0.001).

We constructed sgRNA-expressing plasmids that encode 1, 2, 3 or 6 unique sgRNA_dual-MTS for labeling non-repetitive regions of the *MUC4* gene (sgMUC4_dual-MTS) with the DNA-targeting spacer sequences selected from the pool of 73 *MUC4*-targeting sgRNAs (4) previously used for FP-based CRISPR imaging (Supplementary Tables S1–S4). Following co-transfection of the modified sgRNAs together with dCas9 and BFP (as a transfection control) in HeLa cells, nuclear delivery of ATTO550-labeled FRET donor MB and ATTO647N-labeled FRET acceptor MB was achieved via nucleofection. As a control, cells were also transfected with sgRNA_dual-MTS carrying a nonsense spacer sequence (sgControl_dual-MTS) and nucleofected with the MBs. It was hypothesized that if the dual-MTS does not interfere with sgRNA-guided binding of dCas9 to the target locus, hybridization of both donor and acceptor MBs to the dual-MTS leading to FRET should illuminate the *MUC4* locus as a bright spot when viewed by widefield fluorescence microscopy. It was found that, 24 h after MB delivery, bright spots, ranging from 1–6 copies, could be detected in 41% and 39% of the cells with 6 and 3 sgMUC4_dual-MTS, respectively (Figure 2). The average (± S.E.) spot number was 2.54 ± 0.13 and 2.45 ± 0.11 in cells with six and three sgRNAs, respectively, consistent with previously-reported values for the *MUC4* locus in HeLa cells (4,25). By contrast, very few spots were detectable in cells transfected with two or one sgRNA, similar to results seen with sgControl_dual-MTS. Since bright spots were present in cells with sgMUC4_dual-MTS but to a very low extent in cells with the control sgRNA, it appears that the observed spots arise due to MB hybridization to the dCas9-sgRNA complexes bound to the *MUC4* locus, rather than to free sgRNAs or dCas9-sgRNA complexes. Supporting this, analogous experiments performed in U2OS cells also showed that CRISPR/dual-FRET MB can illuminate the *MUC4* non-repetitive regions with three unique sgMUC4_dual-MTS but not with sgControl_dual-MTS (Supplementary Figure S1A and B). Additionally, dual-FRET MB signals were very close to the cellular background in cells with three unmodified sgRNAs (lacking the dual-MTS) targeting the same *MUC4* non-repetitive regions (sgMUC4) (Supplementary Figure S2), confirming that spots seen in cells with three unique sgMUC4_dual-MTS resulted from specific MB hybridization to the dual-MTS. Evidence that CRISPR/dual-FRET MB can indeed label non-repetitive regions of *MUC4* with three unique sgRNAs came from co-labeling experiments using dCas9 fused to EGFP (dCas9-EGFP) and an unmodified sgRNA (lacking dual-MTS) targeting repetitive regions of sufficient size, necessary for detection (4), on the same chromosome (Figure 3A and B). Specifically, the localization and motion of the CRISPR/dual-FRET MB signals were in high agreement with those of dCas9-EGFP when the unmodified sgRNA recruited multiple dCas9-EGFP to the repetitive regions within the *MUC4* locus, but not when dCas9-EGFP was targeted to more distant repetitive regions (Figure 3C–E, Supplementary Figures S1C–F, S3 and Supplementary Movies S1–S4). Furthermore, CRISPR/dual-FRET MB could also illuminate non-repetitive regions of the *MUC1* gene and an intergenic DNA region (IGR) when using three unique *MUC1*-targeting sgRNA_dual-MTS (sgMUC1_dual-MTS) or IGR-targeting sgRNA_dual-MTS (sgIGR_dual-MTS), respectively (Supplementary Figures S4, S5 and Supplementary Movies S5-S8). Notably, detection efficiency was highly dependent on the sgRNA selection, as a second set of sgRNAs yielded much lower detection efficiency than the primary set (Set I) for each locus (Supplementary Figure S6), presumably because not all genomic regions are equally accessible to dCas9-sgRNA complexes due to differences in topological complexity or presence of DNA-binding proteins. Thus, it is necessary to screen for sgRNAs that can yield detection efficiency much above the background level (i.e. 2.3% of cells with nonspecific spots when expressing sgControl_dual-MTS, see Figure 2B). Finally, a lower signal-to-noise ratio was achieved and fewer genomic loci were detected when single MBs were used to image the *MUC4* loci relative to dual-FRET MBs (Supplementary Figure S7), suggesting that in the context of genomic imaging, the dual-FRET MB approach is more sensitive than the single-MB approach, as seen in previous studies when the two approaches were compared in the context of RNA imaging (15,16). Collectively, these findings indicate that CRISPR/dual-FRET MB, when used in conjunction with widefield fluorescence microscopy, can illuminate non-repetitive genomic regions with as few as three unique sgRNAs.

### Studying the dynamics of non-repetitive genomic loci using CRISPR/dual-FRET MB

**Figure 4.**
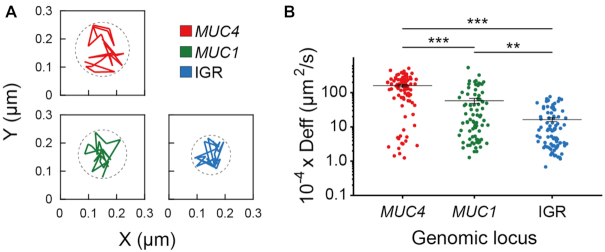
Dynamics of single *MUC4, MUC1* and IGR loci revealed by CRISPR/dual-FRET MBs with three unique sgRNA_dual-MTS. HeLa cells expressing dCas9 and three unique sgMUC4_dual-MTS, sgMUC1_dual-MTS or sgIGR_dual-MTS (Set I) were nucleofected with dual-FRET MBs. Fluorescence microscopy images were taken at 24 h post-nucleofection. (**A**) Representative trajectories of single loci acquired over a 0.5 s time window. Dotted circles indicate the range of motion. (**B**) Scatter plots of the diffusion coefficients (D_eff_) of single loci. Total trajectories of *MUC4* (89 trajectories), *MUC1* (80 trajectories), and IGR (88 trajectories) were analyzed from 81, 68 and 75 cells, respectively. All data represent mean ± S.E. of three independent experiments. Asterisks indicate *P*-values (** *P* < 0.01, *** *P* < 0.001).

Chromatin is highly dynamic and subject to remodeling (26). Therefore, a technique capable of capturing the movement of a specific genomic locus with high spatiotemporal resolution should benefit in-depth characterization of genome architectures in real time. Having successfully demonstrated the ability of CRISPR/dual-FRET MB for illuminating non-repetitive loci with three unique sgRNAs, we next evaluated the capacity of this strategy for imaging chromatin dynamics. To test this, we illuminated the non-repetitive regions of *MUC4, MUC1*, and IGR in HeLa cells using CRISPR/dual-FRET MB with three sgMUC4_dual-MTS, sgMUC1_dual-MTS and sgIGR_dual-MTS (Set I, see Supplementary Figure S6), respectively. Interestingly, single-particle tracking analysis revealed that while all of the detected loci displayed diffusive behavior (α < 1.2), the three different loci displayed unique ranges of motion as well as diffusion coefficients (Figure 4 and Supplementary Movies S9–S11), presumably reflecting their differences in transcription activities and local environment (27). We should emphasize that the three sgRNAs used for imaging, designed to target across ∼770 bp in *MUC4*, ∼300 bp in *MUC1* and ∼220 bp in IGR, were chosen based on protospacer adjacent motif (PAM) availability and spacer specificity. Our CRISPR/dual-FRET MB should also enable other non-repetitive regions of similar or smaller size to be visualized, offering the opportunity for high-definition, live-cell monitoring of genome architectures.

### Conclusion

In summary, we have combined CRISPR and dual-FRET MBs to develop the CRISPR/dual-FRET MB system that can be used to measure the dynamics of non-repetitive regions of distinct genomic loci in the human genome with as few as three unique sgRNAs. It should be noted while non-repetitive genomic regions have previously been visualized via FP-based CRISPR imaging approaches by diffraction-limited fluorescence microscopy (4,6–8), with one sensitive system that employs 4 unique sgRNAs targeting across ∼3800 bp within *MUC4* reporting the presence of 2.2 spots per cell on average (6), each sgRNA was extensively modified to contain 16 MS2 aptamers for tagging 32 copies of MS2-coat protein fused to FP (MCP-FP), thus raising concerns of whether the labeled loci exhibit normal physiological activities. Additionally, the need to append multiple copies of the aptamer in tandem can complicate cloning, as plasmids encoding multiple tandem repeats are susceptible to recombination. Furthermore, dCas9-sgRNA complexes tagged by multiple FPs can be highly susceptible to aggregation, leading to generation of high-intensity nonspecific puncta that can be misinterpreted as genomic loci (10). Given that the total mass of a single CRISPR/dual-FRET MB imaging complex (∼242 kDa) is roughly 14% of the total mass of a single imaging complex of the MS2-based system carrying 32 MCP-FPs (∼1.76 MDa) (Supplementary Table S7) and both systems exhibit similar detection efficiencies for *MUC4* non-repetitive regions, we concluded that CRISPR/dual-FRET MB is a highly sensitive platform for live-cell imaging of non-repetitive genomic loci with the ability to provide the most accurate reflection of normal chromatin dynamics to date.

We should also emphasize that while the CRISPR/dual-FRET MB technology exhibits much greater sensitivity for detecting genomic loci than its parental approach that employs a single MB, it might be less applicable in studies where multiple genomic loci must be visualized simultaneously, since the new technology entails two optically distinct MBs to elicit a FRET signal. We envision the use of more advanced optical techniques, such as super-resolution imaging (28) and multispectral imaging (29), and incorporation of more advanced fluorophore/quencher pairs may expand the versatility of CRISPR/dual-FRET MB, enabling studies of both short- and long-range genome organizations, and their physiological roles, over a broad range of spatial and temporal scales.

## Supplementary Material

gkz752_Supplemental_Files
