# Supplementary material for: CRISPR/dual-FRET molecular beacon for sensitive live-cell imaging of non-repetitive genomic loci
Source: Nucleic Acids Res. 2019 Aug 31;47(20):e131. doi: 10.1093/nar/gkz752 (PMC6847002; doi:10.1093/nar/gkz752)
Supplement: gkz752_Supplemental_Files [file gkz752_supplemental_files.zip › MSQ et al NAR-01180-Met-G-2019.R1 Supplementary Materials.pdf]

SUPPLEMENTARY MATERIALS

CRISPR/Dual-FRET Molecular Beacon for Sensitive Live-Cell Imaging of Non-Repetitive Genomic Loci

Shiqi Mao[1], Yachen Ying[1], Xiaotian Wu[1,2], Christopher J. Krueger[1, 3] and Antony K. Chen[1,*]

[1]Department of Biomedical Engineering, College of Engineering, Peking University, Beijing 100871, China

[2]School of Life Sciences, Peking University, Beijing 100871, China

[3]Wallace H Coulter Department of Biomedical Engineering, Georgia Institute of Technology, Atlanta, GA 30332, USA

Supplementary Figure Legends

Figure S1. Visualizing non-repetitive regions of *MUC4* in U2OS cells using CRISPR/dual-FRET MB. (A, B) Detection of non-repetitive regions of *MUC4* in cells with 3 sgMUC4_dual-MTS (Set I, see Supplementary Table S4) or sgControl_dual-MTS. (A) Representative maximum intensity projection images of the dual-FRET MBs in cells. (B) The distribution of spot number in the cells from (A). The inset shows detection efficiency. n = 300 cells from three independent experiments for each condition. (C-F) Assessment of co-labeling between CRISPR/dual-FRET MB with 3 sgMUC4_dual-MTS (Set I) and dCas9-EGFP with an unmodified sgRNA targeting highly-repetitive genomic regions (sgRNA-rep) on the same chromosome in cells. (C) Schematic showing the position of the non-repetitive regions (NRR) and the repetitive regions (RR) proximal or distal to NRR on chromosome 3 (Chr3) selected for labeling. (D) Representative maximum intensity projection images of dual-FRET MB and dCas9-EGFP signals in fixed cells. Inset shows colocalization of the two signals when dCas9-EGFP labels proximal RR through sgRNA-rep. (E) The percentage of dual-FRET MB signals colocalizing with dCas9-EGFP signals (%Colocalization dual-FRET MB) representing proximal or distal RR on a cell-by-cell basis. n = 33 cells for both conditions. (F) The correlation coefficient between dual-FRET MB and dCas9-EGFP signals (co-movement coefficient) in living cells. 18 trajectories of each signal were analyzed for proximal RR (from 17 cells) and 23 trajectories of each signal were analyzed for distal RR (from 9 cells). Note that co-movement coefficient of 1 indicates perfect co-movement. All data represent mean ± S.E. of three independent experiments. Asterisks indicate P-values (*** $P < 0.001$). Scale bar, 10 µm.

Figure S2. CRISPR/dual-FRET MB signals in HeLa cells with 3 unique *MUC4*-targeting sgRNAs lacking the dual-MTS (sgMUC4). HeLa cells transfected with 3 unique sgMUC4 carrying the same spacer sequences as the 3 unique sgMUC4_dual-MTS (Set I, see Supplementary Table S4) were nucleofected with dual-FRET MBs and then imaged. (A) Representative maximum intensity projection image of the dual-FRET MBs in cells. The dashed line outlines the nucleus. (B) The distribution of spot number in the cells from (A), plotted together with the distribution of spot number in cells with 3 sgMUC4_dual-MTS as in Figure 2B. The inset shows detection efficiency. n = 300 cells from three independent experiments for each condition.

Figure S3. Representative maximum intensity projection FISH images showing large spatial separation between highly-repetitive genomic regions and the *MUC4* loci that are separated by a linear distance of 118 Mb as observed in Figure 3C. HeLa cells transfected with dCas9, sgRNA-rep targeting Distal RR and sgRNA-rep targeting Proximal RR (See Supplementary Table S5 and Figure 3B) were subjected to FISH processing using Distal RR-targeting FISH probes (ATTO488-labeled) and Proximal RR-targeting FISH probes (TAMRA-labeled) (See Materials and Methods for FISH processing procedures). DAPI stains the nucleus. Scale bar, 10 µm.

Figure S4. Visualizing non-repetitive regions of *MUC1* in HeLa cells using CRISPR/dual-FRET MB. (A, B) Detection of non-repetitive regions of *MUC1* in cells with 3 sgMUC1_dual-MTS (Set I, see Supplementary Table S4). (A) Representative maximum intensity projection image of the dual-FRET MBs in cells. (B) The distribution of spot number in the cells from (A), plotted together with the distribution of spot number in cells with sgControl_dual-MTS as in Figure 2B. The inset shows detection efficiency. n = 300 cells from three independent experiments for each condition. (C-F) Assessment of co-labeling between CRISPR/dual-FRET MBs with 3 sgMUC1_dual-MTS (Set I) and dCas9-EGFP with an unmodified sgRNA targeting highly-repetitive genomic regions (sgRNA-rep) on the same chromosome in cells. (C) Schematic showing the position of the NRR and the RR proximal or distal to NRR on chromosome 1 (Chr1) selected for labeling. (D) Representative maximum intensity projection images of dual-FRET MB and dCas9-EGFP signals in fixed cells. Inset shows colocalization of the two signals when dCas9-EGFP labels proximal RR through sgRNA-rep. (E) The percentage of dual-FRET MB signals colocalizing with dCas9-EGFP signals (%Colocalization dual-FRET MB) representing proximal or distal RR on a cell-by-cell basis. n = 32 cells for proximal RR and 30 cells for distal RR. (F) The correlation coefficient between dual-FRET MB and dCas9-EGFP signals (co-movement coefficient) in living cells. 15 trajectories of each signal were analyzed for proximal RR (from 15 cells) and 15 trajectories of each signal were analyzed for distal RR (from 10 cells). Note that co-movement coefficient of 1 indicates perfect co-movement. All data represent mean ± S.E. of three independent experiments. Asterisks indicate *P*-values (*** $P < 0.001$). Scale bar, 10 μm.

Figure S5. Visualizing non-repetitive regions of IGR in HeLa cells using CRISPR/dual-FRET MB. (A,B) Detection of non-repetitive regions of IGR in cells with 3 sgIGR_dual-MTS (Set I, see Supplementary Table S4). (A) Representative maximum intensity projection image of the dual-FRET MBs in cells. (B) The distribution of spot number in the cells from (A), plotted together with the distribution of spot number in cells with sgControl_dual-MTS as in Figure 2B. The inset shows detection efficiency. n = 300 cells from three independent experiments for each condition. (C-F) Assessment of co-labeling between CRISPR/dual-FRET MBs with 3 sgIGR_dual-MTS (Set I) and dCas9-EGFP with an unmodified sgRNA targeting highly-repetitive genomic regions (sgRNA-rep) on the same chromosome in cells. (C) Schematic showing the position of the NRR and the RR proximal or distal to NRR on chromosome 19 (Chr19) selected for labeling. (D) Representative maximum intensity projection images of dual-FRET MB and dCas9-EGFP signals in fixed cells. Inset shows colocalization of the two signals when dCas9-EGFP labels proximal RR through sgRNA-rep. (E) The percentage of dual-FRET MB signals colocalizing with dCas9-EGFP signals (%Colocalization dual-FRET MB) representing proximal or distal RR on a cell-by-cell basis. n = 30 cells for proximal RR and 50 cells for distal RR. (F) The correlation coefficient between dual-FRET MB and dCas9-EGFP signals (co-movement coefficient) in living cells. 15 trajectories of each signal were analyzed for proximal RR (from 15 cells) and 15 trajectories of each signal were analyzed for distal RR (from 13 cells). Note that co-movement coefficient of 1 indicates perfect co-movement. All data

represent mean ± S.E. of three independent experiments. Asterisks indicate *P*-values (*** $P < 0.001$). Scale bar, 10 μm.

Figure S6. The effect of sgRNA selection on CRISPR/dual-FRET MB-based labeling of non-repetitive genomic regions. HeLa cells transfected with a second set (Set II, see Supplementary Table S4) of 3 sgMUC4_dual-MTS, 3 sgMUC1_dual-MTS, or 3 sgIGR_dual-MTS were nucleofected with donor and acceptor MBs and then imaged for the presence of bright spots. The distribution of the spot number in the cells is shown, plotted together with the distribution of the spot number for Set I as in Figure 2B and Supplementary Figure S4B and S5B. The inset shows detection efficiency. n = 300 cells from three independent experiments for each condition.

Figure S7. CRISPR imaging methods incorporating single MBs have reduced capacity in labeling non-repetitive genomic regions compared with CRISPR imaging methods incorporating dual-FRET MBs (CRISPR/dual-FRET MB). HeLa cells transfected with 3 sgMUC4_dual-MTS (Set I, see Supplementary Table S4) were nucleofected with acceptor MBs only and then imaged for the presence of bright spots. (A) The distribution of the spot number in the cells is shown, plotted together with the distribution of the spot number for CRISPR/dual-FRET MB as in Figure 2B. The inset shows detection efficiency. n = 300 cells from three independent experiments for each condition. (B) Signal-to-noise ratios of the spots detected by single MBs (n=18 spots from 17 cells) and dual-FRET MBs (n =22 spots from 21 cells). Data represent mean ± S.E. Asterisks indicate *P*-values (*** $P < 0.001$).

Supplementary Table Legends

Table S1. Sequences of the sgRNA_dual-MTS, sgMUC4 and sgRNA-rep scaffolds used in this study. For sgRNA_dual-MTS, the dual-MTS sequence is underlined and additional sequences flanking the dual-MTS are italicized.

Table S2. Spacer sequence and target region information of the sgRNAs used for labeling non-repetitive regions.

Table S3. Sequences of PCR primers used for constructing the backbone plasmids of the sgRNAs used for labeling non-repetitive regions via PCR-mediated site directed mutagenesis.

Table S4. Different sgRNAs used for single or multiplexed sgRNA expression.

Table S5. Spacer sequence and target region information of the sgRNAs used for labeling repetitive regions.

Table S6. Sequences of PCR primers used for constructing the subcloning vectors of the sgRNAs used for labeling repetitive regions via PCR-mediated site directed mutagenesis.

Table S7. Calculation of total mass of the dCas9-sgRNA imaging complex of the CRISPR/dual-FRET MB system and the MS2-based CRISPR imaging system carrying 32 MCP-FPs used in reference (6).

Supplementary Movie Legends

Movie S1. Representative movie of non-repetitive regions of *MUC4* revealed by CRISPR/dual-FRET MB with 3 sgMUC4_dual-MTS (red) and highly-repetitive regions of *MUC4* revealed by dCas9-EGFP with sgRNA-rep (proximal) (green) in the nucleus of HeLa cells. The acquisition rate was 10 frames per second (fps). The video includes 100 frames (a total time of 10 seconds) and the play rate is 10 fps.

Movie S2. Representative movie of non-repetitive regions of *MUC4* revealed by CRISPR/dual-FRET MB with 3 sgMUC4_dual-MTS (red) and highly-repetitive regions distal to *MUC4* revealed by dCas9-EGFP with sgRNA-rep (distal) (green) in the nucleus of HeLa cells. The acquisition rate was 10 fps. The video includes 100 frames (a total time of 10 seconds) and the play rate is 10 fps.

Movie S3. Representative movie of non-repetitive regions of *MUC4* revealed by CRISPR/dual-FRET MB with 3 sgMUC4_dual-MTS (red) and highly-repetitive regions of *MUC4* revealed by dCas9-EGFP with sgRNA-rep (proximal) (green) in the nucleus of U2OS cells. The acquisition rate was 10 fps. The video includes 100 frames (a total time of 10 seconds) and the play rate is 10 fps.

Movie S4. Representative movie of non-repetitive regions of *MUC4* revealed by CRISPR/dual-FRET MB with 3 sgMUC4_dual-MTS (red) and highly-repetitive regions distal to *MUC4* revealed by dCas9-EGFP with sgRNA-rep (distal) (green) in the nucleus of U2OS cells. The acquisition rate was 10 fps. The video includes 100 frames (a total time of 10 seconds) and the play rate is 10 fps.

Movie S5. Representative movie of non-repetitive regions of *MUC1* revealed by CRISPR/dual-FRET MB with 3 sgMUC1_dual-MTS (red) and highly-repetitive regions of *MUC1* revealed by dCas9-EGFP with sgRNA-rep (proximal) (green) in the nucleus of HeLa cells. The acquisition rate was 10 fps. The video includes 100 frames (a total time of 10 seconds) and the play rate is 10 fps.

Movie S6. Representative movie of non-repetitive regions of *MUC1* revealed by CRISPR/dual-FRET MB with 3 sgMUC1_dual-MTS (red) and highly-repetitive regions distal to *MUC1* revealed by dCas9-EGFP with sgRNA-rep (distal) (green) in the nucleus of HeLa cells. The acquisition rate was 10 fps. The video includes 100 frames (a total time of 10 seconds) and the play rate is 10 fps.

Movie S7. Representative movie of non-repetitive regions of IGR revealed by CRISPR/dual-FRET MB with 3 sgIGR_dual-MTS (red) and highly-repetitive regions of IGR revealed by dCas9-EGFP with sgRNA-rep (proximal) (green) in the nucleus of HeLa cells. The acquisition rate was 10 fps. The video includes 100 frames (a total time of 10 seconds) and the play rate is 10 fps.

Movie S8. Representative movie of non-repetitive regions of IGR revealed by CRISPR/dual-FRET MB with 3 sgIGR_dual-MTS (red) and highly-repetitive regions distal to IGR revealed by dCas9-EGFP with sgRNA-rep (distal) (green) in the nucleus of HeLa cells. The acquisition rate was 10 fps. The video includes 100 frames (a total time of 10 seconds) and the play rate is 10 fps.

Movie S9. Representative movie of non-repetitive regions of *MUC4* revealed by CRISPR/dual-FRET MBs with 3 sgMUC4_dual-MTS in the nucleus of HeLa cells. The acquisition rate was 50 fps. The video includes 250 frames (a total time of 5 seconds), and the play rate is 50 fps.

Movie S10. Representative movie of non-repetitive regions of *MUC1* revealed by CRISPR/dual-FRET MBs with 3 sgMUC1_dual-MTS in the nucleus of HeLa cells. The acquisition rate was 50 fps. The video includes 250 frames (a total time of 5 seconds), and the play rate is 50 fps.

Movie S11. Representative movie of non-repetitive regions of IGR revealed by CRISPR/dual-FRET MBs with 3 sgIGR_dual-MTS in the nucleus of HeLa cells. The acquisition rate was 50 fps. The video includes 250 frames (a total time of 5 seconds), and the play rate is 50 fps.

Supplementary Figure S1.

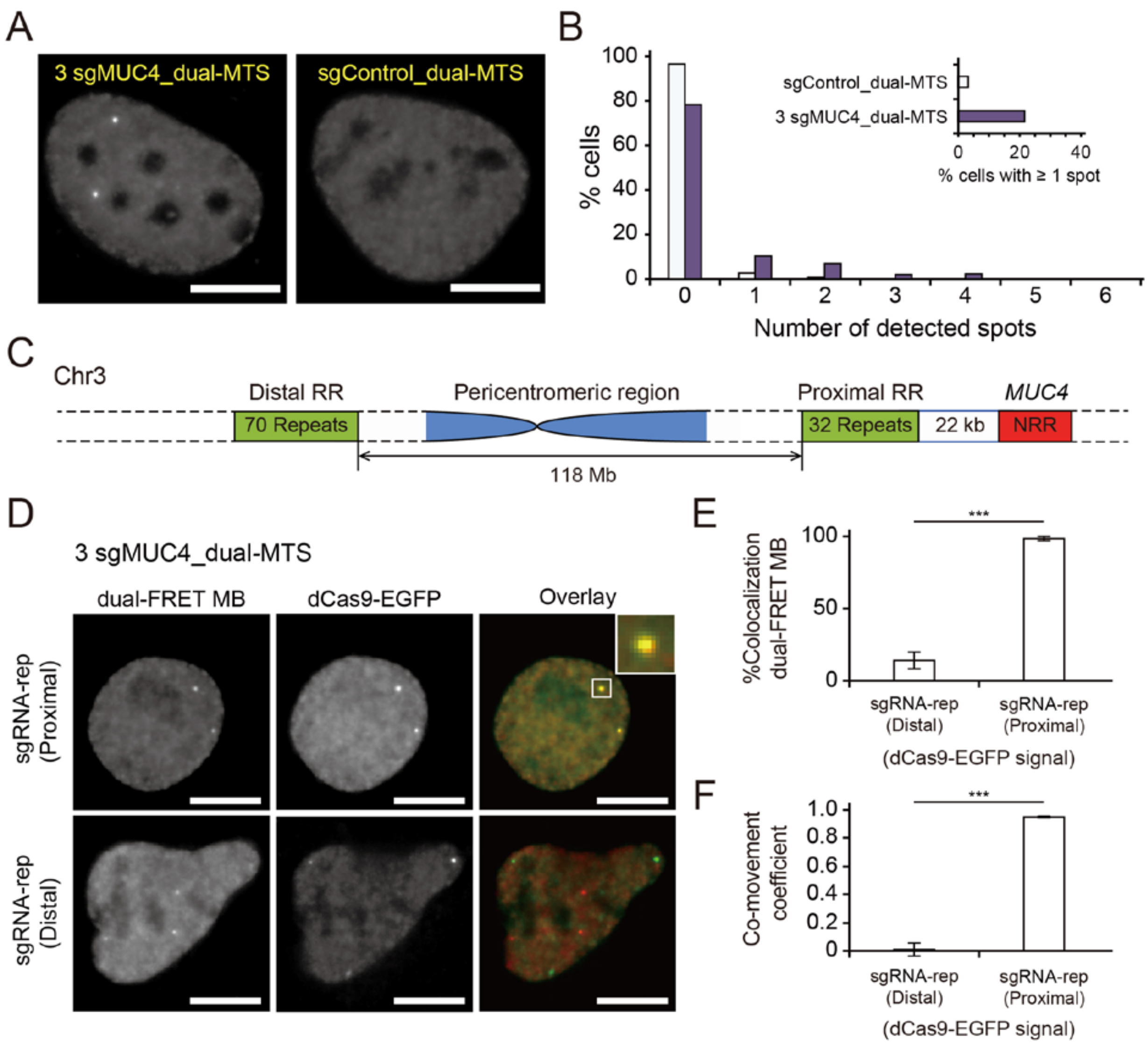

Supplementary Figure S2

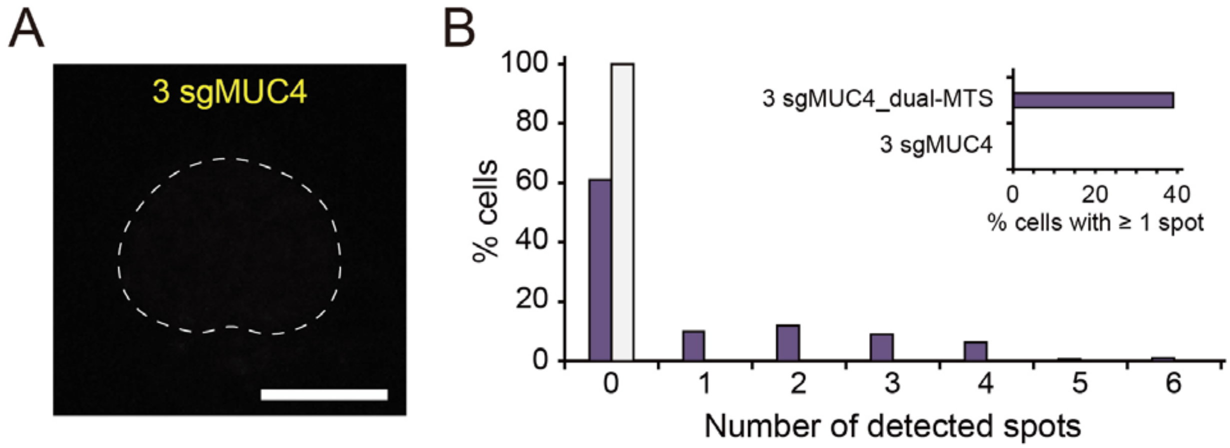

Supplementary Figure S3.

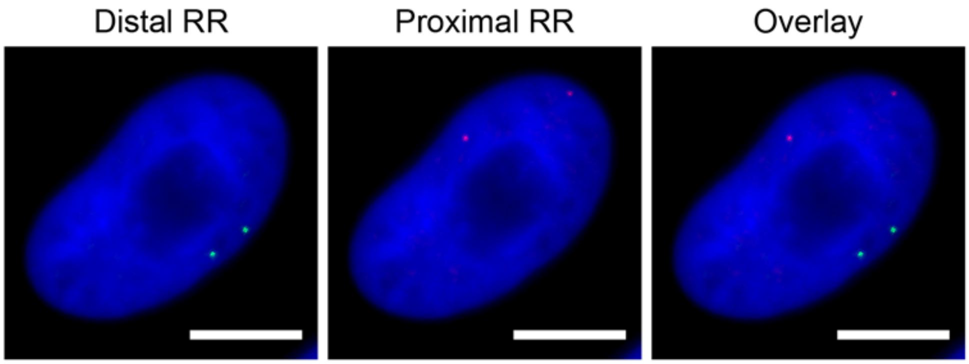

Supplementary Figure S4.

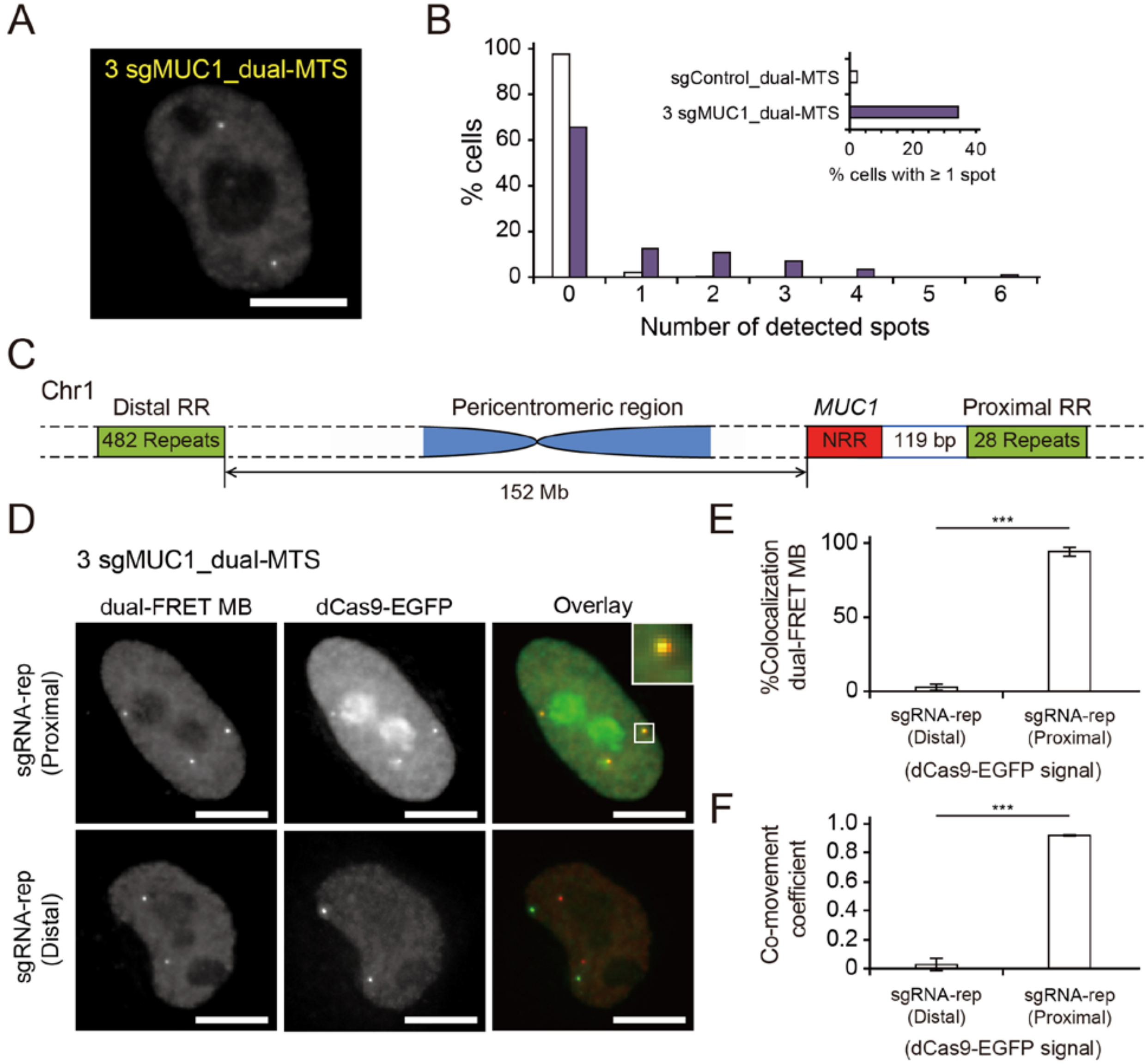

Supplementary Figure S5.

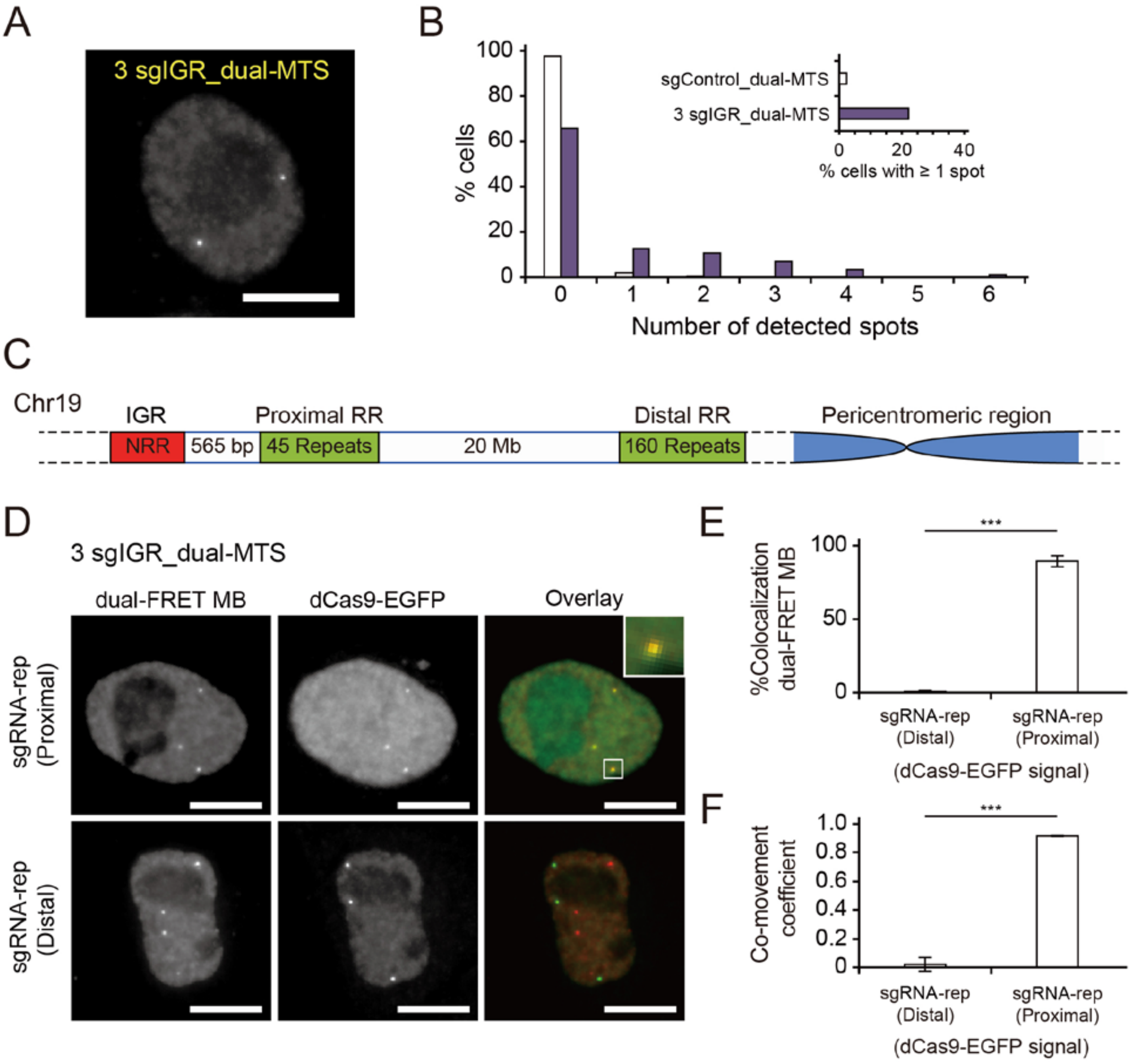

Supplementary Figure S6.

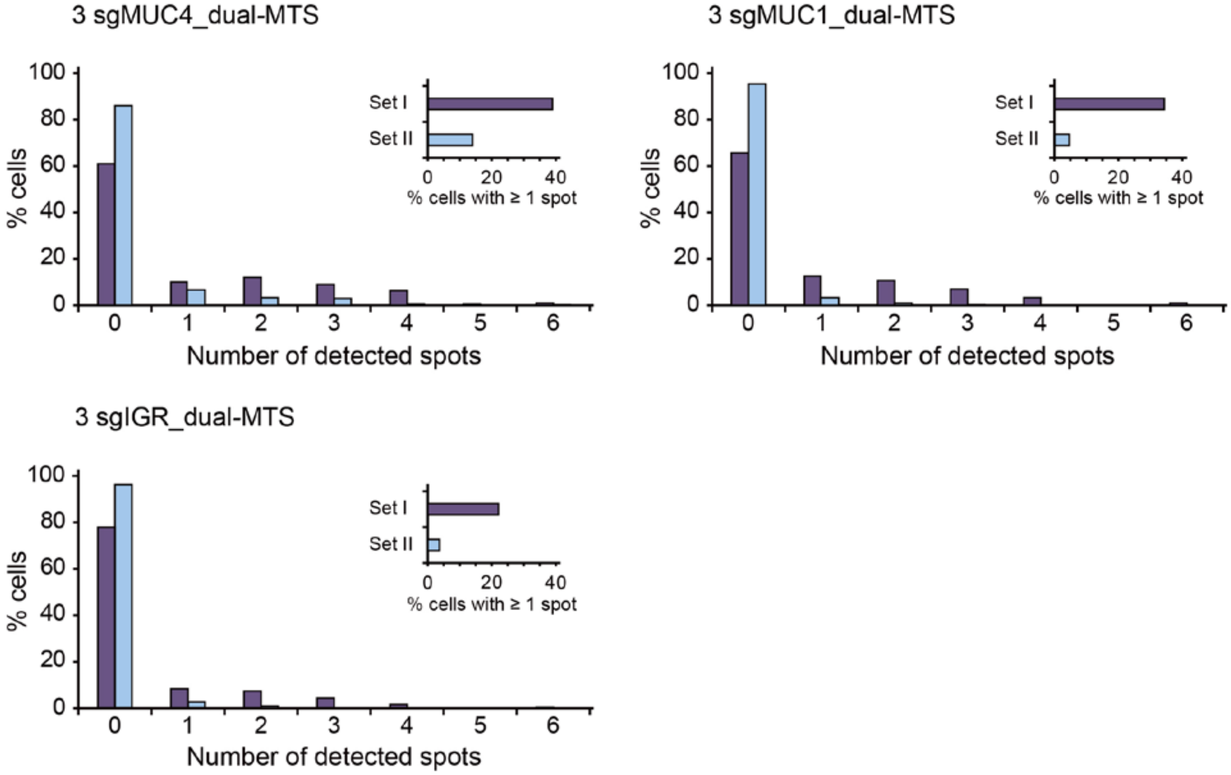

Supplementary Figure S7.

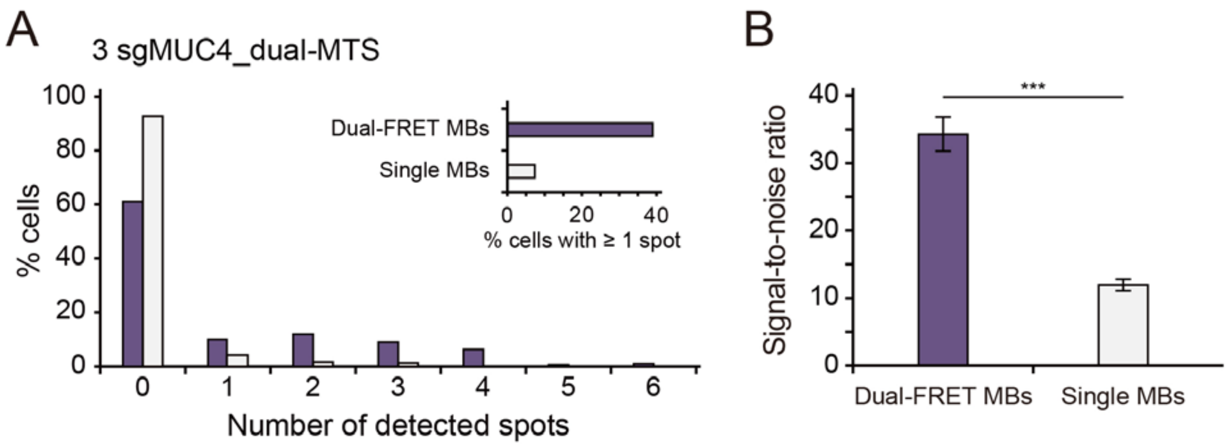

Supplementary Table S1

**sgRNA scaffold name**	**sgRNA scaffold sequence**
sgRNA_dual-MTS	GTTTGAGAGCTATGCTGGAAACAGCATAGCAAGTTCAAATAAGGCTAGTCCGTTATCAACTT*GGCCCCGGAGCAGAACGA*CAGGAGTTGTGTTTGTGGACGAAGCAAGCTCAGTCACGACATCACTTACGCT*AGCCTGCAGTCTGCTCCGGGGCC*AGTGGCACCGAGTCGGTGCTTTTTTT
sgMUC4	GTTTGAGAGCTATGCTGGAAACAGCATAGCAAGTTCAAATAAGGCTAGTCCGTTATCAACTTGAAAAAGTGGCACCGAGTCGGTGCTTTTTTT
sgRNA-rep	GTTTAAGAGCTATGCTGGAAACAGCATAGCAAGTTTAAATAAGGCTAGTCCGTTATCAACTTGAAAAAGTGGCACCGAGTCGGTGCTTTTTTT

Supplementary Table S2

sgRNA name	sgRNA scaffold name	Target region			sgRNA spacer sequence
		Chromosome number	Position		
			Start	End	
sgMUC4_dual-MTS_1	sgRNA_dual-MTS	3	195810880	195810901	GTACACCCTTGTG TACAGAGCT
sgMUC4_dual-MTS_2	sgRNA_dual-MTS	3	195811461	195811482	GAAGAGTGGAGG CCGTGCGCGG
sgMUC4_dual-MTS_3	sgRNA_dual-MTS	3	195810713	195810734	GCAAGCAAGGGA AGCGACAAGG
sgMUC4_dual-MTS_4	sgRNA_dual-MTS	3	195808506	195808526	GTAAAGTAGAAA AGGCATAAA
sgMUC4_dual-MTS_5	sgRNA_dual-MTS	3	195808147	195808168	GAACCCGGAATG GCACTTGTGT
sgMUC4_dual-MTS_6	sgRNA_dual-MTS	3	195807221	195807243	GCTCGCCTCGGCT CCCAAAGTGC
sgMUC1_dual-MTS_1	sgRNA_dual-MTS	1	155188041	155188055	GATTTATAAACA AGG
sgMUC1_dual-MTS_2	sgRNA_dual-MTS	1	155188219	155188232	GTTTAATTCCTCT C
sgMUC1_dual-MTS_3	sgRNA_dual-MTS	1	155188330	155188344	GCACTCACCATA GCA
sgMUC1_dual-MTS_4	sgRNA_dual-MTS	1	155187481	155187495	GTGTTCTGGTTGC GC
sgMUC1_dual-MTS_5	sgRNA_dual-MTS	1	155187830	155187842	GTACAATTGACT C
sgMUC1_dual-MTS_6	sgRNA_dual-MTS	1	155187719	155187731	GACGTCAGCGGT G
sgIGR_dual-MTS_1	sgRNA_dual-MTS	19	380053	380071	GTCTCTCGACTGT TGCGTT
sgIGR_dual-MTS_2	sgRNA_dual-MTS	19	380125	380137	GTGGACACCGTT G

sgIGR_dual-MTS_3	sgRNA_dual-MTS	19	380259	380270	GCGCTCCATGTA
sgIGR_dual-MTS_4	sgRNA_dual-MTS	19	380333	380346	GCTTCAGTTTAATA
sgIGR_dual-MTS_5	sgRNA_dual-MTS	19	380381	380392	GAAGCCACGAAT
sgIGR_dual-MTS_6	sgRNA_dual-MTS	19	380473	380486	GATGAGTGCTGGCT
sgControl_dual-MTS	sgRNA_dual-MTS	-	-	-	GAGCTGCACGCTGCCGTC
sgMUC4 _1	sgMUC4	3	195810880	195810901	GTACACCCTTGTGTACAGAGCT
sgMUC4_ 2	sgMUC4	3	195811461	195811482	GAAGAGTGGAGGCCGTGCGCGG
sgMUC4_ 3	sgMUC4	3	195810713	195810734	GCAAGCAAGGGAAGCGACAAGG

Supplementary Table S3

sgRNA name	Forward primers	Reverse primers
sgMUC4_dual-MTS_1	GTACACCCTTGTGTACAGAGCTGTT TGAGAGCTATGCTGGAAACA	GGTGTTTCGTCCTTTCCACAAGATA
sgMUC4_dual-MTS_2	GAAGAGTGGAGGCCGTGCGCGGGT TTGAGAGCTATGCTGGAAACA	GGTGTTTCGTCCTTTCCACAAGATA
sgMUC4_dual-MTS_3	GCAAGCAAGGGAAGCGACAAGGG TTTGAGAGCTATGCTGGAAACA	GGTGTTTCGTCCTTTCCACAAGATA
sgMUC4_dual-MTS_4	GTAAAGTAGAAAAGGCATAAAGTT TGAGAGCTATGCTGGAAACA	GGTGTTTCGTCCTTTCCACAAGATA
sgMUC4_dual-MTS_5	GAACCCGGAATGGCACTTGTGTGTT TGAGAGCTATGCTGGAAACA	GGTGTTTCGTCCTTTCCACAAGATA
sgMUC4_dual-MTS_6	GCTCGCCTCGGCTCCCAAAGTGCGT TTGAGAGCTATGCTGGAAACA	GGTGTTTCGTCCTTTCCACAAGATA
sgMUC1_dual-MTS_1	GATTTATAAACAAGGGTTTGAGAG CTATGCTGGAAACA	GGTGTTTCGTCCTTTCCACAAGATA
sgMUC1_dual-MTS_2	GTTTAATTCCTCTCGTTTGAGAGCT ATGCTGGAAACA	GGTGTTTCGTCCTTTCCACAAGATA
sgMUC1_dual-MTS_3	GCACTCACCATAGCAGTTTGAGAG CTATGCTGGAAACA	GGTGTTTCGTCCTTTCCACAAGATA
sgMUC1_dual-MTS_4	GTGTTCTGGTTGCGCGTTTGAGAGC TATGCTGGAAACA	GGTGTTTCGTCCTTTCCACAAGATA
sgMUC1_dual-MTS_5	GTACAATTGACTCGTTTGAGAGCTA TGCTGGAAACA	GGTGTTTCGTCCTTTCCACAAGATA
sgMUC1_dual-MTS_6	GACGTCAGCGGTGGTTTGAGAGCT ATGCTGGAAACA	GGTGTTTCGTCCTTTCCACAAGATA
sgIGR_dual-MTS_1	GTCTCTCGACTGTTGCGTTGTTTGA GAGCTATGCTGGAAACA	GGTGTTTCGTCCTTTCCACAAGATA
sgIGR_dual-MTS_2	GTGGACACCGTTGGTTTGAGAGCTA TGCTGGAAACA	GGTGTTTCGTCCTTTCCACAAGATA
sgIGR_dual-MTS_3	GCGCTCCATGTAGTTTGAGAGCTAT GCTGGAAACA	GGTGTTTCGTCCTTTCCACAAGATA
sgIGR_dual-MTS_4	GCTTCAGTTTAATAGTTTGAGAGCT ATGCTGGAAACA	GGTGTTTCGTCCTTTCCACAAGATA
sgIGR_dual-MTS_5	GAAGCCACGAATGTTTGAGAGCTA TGCTGGAAACA	GGTGTTTCGTCCTTTCCACAAGATA
sgIGR_dual-MTS_6	GATGAGTGCTGGCTGTTTGAGAGCT ATGCTGGAAACA	GGTGTTTCGTCCTTTCCACAAGATA
sgControl_dual-MTS	GAGCTGCACGCTGCCGTCGTTTGA GAGCTATGCTGGAAACA	GGTGTTTCGTCCTTTCCACAAGATA

Supplementary Table S4

Target locus	# of unique sgRNAs	Set number	Expression vector	sgRNA combination
MUC4	6	-	pGEM	sgMUC4_dual-MTS_1<br>sgMUC4_dual-MTS_2<br>sgMUC4_dual-MTS_3<br>sgMUC4_dual-MTS_4<br>sgMUC4_dual-MTS_5<br>sgMUC4_dual-MTS_6
MUC4	3	I	pGEM	sgMUC4_dual-MTS_1<br>sgMUC4_dual-MTS_2<br>sgMUC4_dual-MTS_3
MUC4	3	II	pGEM	sgMUC4_dual-MTS_4<br>sgMUC4_dual-MTS_5<br>sgMUC4_dual-MTS_6
MUC4	2	-	pGEM	sgMUC4_dual-MTS_1<br>sgMUC4_dual-MTS_2
MUC4	1	-	pGEM	sgMUC4_dual-MTS_1
MUC1	3	I	pGEM	sgMUC1_dual-MTS_1<br>sgMUC1_dual-MTS_2<br>sgMUC1_dual-MTS_3
MUC1	3	II	pGEM	sgMUC1_dual-MTS_4<br>sgMUC1_dual-MTS_5<br>sgMUC1_dual-MTS_6
IGR	3	I	pGEM	sgIGR_dual-MTS_1<br>sgIGR_dual-MTS_2<br>sgIGR_dual-MTS_3
IGR	3	II	pGEM	sgIGR_dual-MTS_4<br>sgIGR_dual-MTS_5<br>sgIGR_dual-MTS_6
EGFP	1	-	pGEM	sgControl_dual-MTS
MUC4	3	I	pGEM	sgMUC4_1<br>sgMUC4_2<br>sgMUC4_3

Supplementary Table S5

sgRNA name	sgRNA scaffold name	Expression vector	Target region				sgRNA spacer sequence
			Chromosome number	Position		Copies	
				Start	End		
sgRNA-rep (Proximal to *MUC4*-NRR)	sgRNA-rep	pSLQ1661	3	195778912	195788293	32	GTGGCGT GACCTGTG GATGCTG
sgRNA-rep (Distal to *MUC4*-NRR)	sgRNA-rep	pSLQ1661	3	77289276	77296418	70	GACATAAA GTAAAATT GA
sgRNA-rep (Proximal to *MUC1*-NRR)	sgRNA-rep	pSLQ1661	1	155188584	155191956	28	GCTCCACC GCCCCCCC AGCCCA
sgRNA-rep (Distal to *MUC1*-NRR)	sgRNA-rep	pSLQ1661	1	2648592	2777186	482	AGATGCTC ACC
sgRNA-rep (Proximal to IGR-NRR)	sgRNA-rep	pSLQ1661	19	380836	382651	45	AGCAGAT GTAGG
sgRNA-rep (Distal to IGR-NRR)	sgRNA-rep	pSLQ1661	19	20866764	20916281	160	GTGACAG TGAAC

Supplementary Table S6

sgRNA name	Forward primers	Reverse primers
sgRNA-rep (Distal to *MUC4*-NRR)	GACATAAAGTAAAATTGAGTTTAAGAGCTATGCTGGAAACA	CAACAAGGTGGTTCTCCAAGGGATA
sgRNA-rep (Proximal to *MUC1*-NRR)	GCTCCACCGCCCCCCCAGCCCAGTTTAAGAGCTATGCTGGAAACA	CAACAAGGTGGTTCTCCAAGGGATA
sgRNA-rep (Distal to *MUC1*-NRR)	GAGATGCTCACCGTTTAAGAGCTATGCTGGAAACA	CAACAAGGTGGTTCTCCAAGGGATA
sgRNA-rep (Proximal to IGR-NRR)	GAGCAGATGTAGGGTTTAAGAGCTATGCTGGAAACA	CAACAAGGTGGTTCTCCAAGGGATA
sgRNA-rep (Distal to IGR-NRR)	GTGACAGTGAACGTTTAAGAGCTATGCTGGAAACA	CAACAAGGTGGTTCTCCAAGGGATA

Supplementary Table S7

System	dCas9 (Da)	Modified sgRNA			Effector			Total molecular weight of the dCas9-sgRNA complex (Da)
		Average molecular weight per base (Da/nt)	Length (nt)	Total molecular weight (Da)	Unit molecular weight (Da)	Number	Total molecular weight (Da)	
CRISPR/dual-FRET MB	162,000	321.25	181	58,146	11,000 (MB)	2	22,000	242,146
The MS2-based CRISPR imaging system carrying 32 MCP-FPs	162,000	321.25	990	318,038	40,000 (MCP-FP)	32	1,280,000	1,760,038
